# Lung cancer in HIV patients and their parents: A Danish cohort study

**DOI:** 10.1186/1471-2407-11-272

**Published:** 2011-06-25

**Authors:** Frederik N Engsig, Gitte Kronborg, Carsten S Larsen, Gitte Pedersen, Court Pedersen, Jan Gerstoft, Niels Obel

**Affiliations:** 1 Department of Infectious Diseases, Copenhagen University Hospital, Rigshospitalet, Blegdamsvej 9, 2100 Kbh Ø, Denmark; 2 Department of Infectious Diseases, Copenhagen University Hospital, Hvidovre Hospital, Kettegårds Allé 30, 2650 Hvidovre, Denmark; 3 Department of Infectious Diseases, Aarhus University Hospital, Brendstrupgårdsvej, 8200 Aarhus N, Denmark; 4 Department of Infectious Diseases, Aalborg University Hospital, 9000 Aalborg, Denmark; 5 Department of Infectious Diseases, Odense University Hospital, Sønderbouldevard 65, 5000 Odense C, Denmark

**Keywords:** HIV, lung cancer incidence, matched cohort, population controls, parents, immunosuppression

## Abstract

**Background:**

HIV patients are known to be at increased risk of lung cancer but the risk factors behind this are unclear.

**Methods:**

We estimated the cumulative incidence and relative risk of lung cancer in 1) a population of all Danish HIV patients identified from the Danish HIV Cohort Study (n = 5,053) and a cohort of population controls matched on age and gender (n = 50,530) (study period; 1995 - 2009) and 2) their parents (study period; 1969 - 2009). Mortality and relative risk of death after a diagnosis of lung cancer was estimated in both populations.

**Results:**

29 (0.6%) HIV patients vs. 183 (0.4%) population controls were diagnosed with lung cancer in the observation period. HIV patients had an increased risk of lung cancer (adjusted incidence rate ratio (IRR); 2.38 (95% CI; 1.61 - 3.53)). The IRR was considerably increased in HIV patients who were smokers or former smokers (adjusted IRR; 4.06 (95% CI; 2.66 - 6.21)), male HIV patients with heterosexual route of infection (adjusted IRR; 4.19 (2.20 - 7.96)) and HIV patients with immunosuppression (adjusted IRR; 3.25 (2.01 - 5.24)). Both fathers and mothers of HIV patients had an increased risk of lung cancer (adjusted IRR for fathers; 1.31 (95% CI: 1.09 - 1.58), adjusted IRR for mothers 1.35 (95% CI: 1.07 - 1.70)). Mortality after lung cancer diagnose was increased in HIV patients (adjusted mortality rate ratio 2.33 (95%CI; 1.51 - 3.61), but not in the parents. All HIV patients diagnosed with lung cancer were smokers or former smokers.

**Conclusion:**

The risk was especially increased in HIV patients who were smokers or former smokers, heterosexually infected men or immunosuppressed. HIV appears to be a marker of behavioural or family related risk factors that affect the incidence of lung cancer in HIV patients.

## Background

After the introduction of highly active antiretroviral therapy (HAART) HIV has changed from a fatal disease to a chronic condition and well treated HIV patients now have an overall life expectancy close to that of non-HIV infected individuals [1]. Due to immunological recovery, there has been a remarkable decline in AIDS defining cancers whereas the increased risk of certain non-AIDS defining cancers, including lung cancer has persisted in the HIV population [2-6].

Lung cancer is primarily related to tobacco use and the role of HIV infection in the development of lung cancer is uncertain [7]. Several studies have shown that both immunosuppressed patients after allograft organ transplantation and HIV patients are at higher risk of lung cancer [8]. Therefore immunodeficiency and chronic inflammation are proposed to be major risk factors, besides smoking, involved in the lung cancer pathogenesis. Whereas the role of HIV infection in lung cancer development is questionable, most studies show a decreased survival in HIV patients with lung cancer [9-12]. We hypothesized that family related risk factors may be part of the increased risk of lung cancer and mortality and that HIV is a marker for the increased risk independently of the pathogenicity of HIV. We therefore performed a national cohort study comparing the risk of lung cancer and survival among Danish HIV patients, their parents and a cohort of population controls.

## Methods

In the first part of the study we estimate the incidence of lung cancer in 1) HIV patients compared to population controls matched on age and gender and 2) in the parents of the HIV patients compared to the parents of the population controls. In the second part of the study we estimate the mortality of individuals diagnosed with lung cancer in 1) HIV patients compared to population controls matched on age and gender and 2) the parents of the HIV patients compared to the parents of the population controls.

### Setting

Denmark had a population of 5.5 million as of 31 December 2008, with an estimated HIV prevalence of approximately 0.09% in the adult population. Patients with HIV infection are treated in the country's eight specialized medical centres, where they are seen on an outpatient basis at intended intervals of 12 weeks. As HAART is available only at these eight centres almost no HIV patients are treated elsewhere. Antiretroviral treatment is provided free of charge to all HIV-infected residents of Denmark.

### Data sources

We used the unique 10-digit civil registration number assigned to all individuals in Denmark at birth or upon immigration to link the data sources described below [13].

The Danish HIV Cohort study (DHCS) is a population-based prospective nationwide cohort study of all HIV patients 16 years or older at diagnosis and who are treated at Danish HIV centres after 1 January 1995 [14]. The HIV patients are consecutively enrolled, and multiple registrations are avoided through the use of the unique civil registration number. December 31, 2009 the cohort included 5481 Danish residents. Data are updated yearly and includes demographics, smoking status, date of HIV infection, AIDS defining events, date and cause of death and antiretroviral treatment. CD4^+ ^cell counts and HIV-RNA measurements are extracted electronically from laboratory data files. Data on smoking are not adequate in the database. Patients who were registered at least once as consuming tobacco in any quantity were considered smokers or former smokers. We calculated the distribution of smokers or former smokers among Danish HIV infected.

The Danish Civil Registration System (DCRS) was established in 1968 and stores information of vital status, residency as well as immigration and emigration on all Danish residents [13]. Since 1 January 1969 the registry also included identification of parents still alive at this date.

The Danish Cancer Register is a population-based register and contains information on incident cancers diagnosed in Danish Citizens since 1943. Details about registration can be found elsewhere [15]. In 1969 - 2003 cancers were coded according to International Classification of Disease version 7 (ICD-7) and International Classification of Diseases for Oncology 1 (ICD-O-1) with supplement from ICD-O-2 in 1990 - 2003. Since 2004 cancers have been coded according to ICD-O-3 and ICD-10 and data from the period 1978 - 2003 have been converted to ICD-O-3 and ICD-10. We do not have data on cancer staging or treatment from the Danish Cancer Register.

Data on smoking are not available in the Danish national registries.

### Study populations

#### HIV and population control Study populations

**Figure 1:**
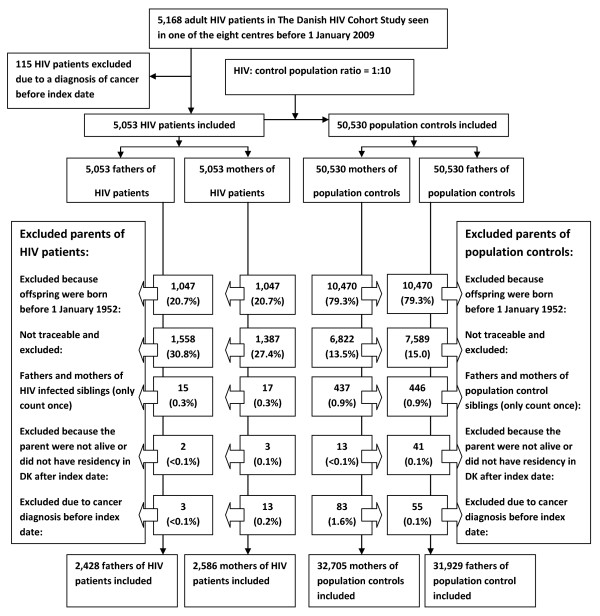
**Summary of the study design**.

In the first part of the study, we included all HIV patients from the Danish HIV Cohort Study without a diagnosis of cancer prior to index date, see Figure 1. The index date was defined as 1 January 1995, the date of the HIV diagnosis or date of immigration, which ever came last. For each of the HIV patients we identified 10 age- and gender matched population control subjects from the DCRS who were alive and living in Denmark at index date of the corresponding HIV patient (refereed to also as the index date of the respective population control) and not diagnosed with a cancer prior to index date.

#### Parent study populations

In the parent study population we included all mothers and fathers of the HIV patients and population controls included who 1) gave birth to a index patient or population control after 1 January 1952, 2) were alive and living in Denmark after 1 January 1969 and 3) who were not diagnosed with cancer prior to the parent index date, see Figure 1. The parent index date was defined as 1 January 1969, date of birth of the included HIV patient/population control or date of registration in DCRS, whichever came last. In case a parent was the father or mother of both an HIV patient and a population control, they were included in both parent populations.

### Outcome

In the first study outcome was time to first diagnosis of lung cancer. We identified all primary lung cancers in the populations using cancer diagnoses as defined by NORDCAN [16]; see cancer definitions in Additional file 1. Lung cancer was defined according to NORDCAN: 1620, 1621, 1628 in ICD-7 and C34 in ICD-10. In order to investigate if there was any difference in the histopathological distribution of lung cancer perhaps due to immunodeficiency we grouped the lung cancers into five groups following the 1999 WHO classification [17] categorized by the following ICD-O-3 codes; 1) squamous cell carcinoma (M80503 - 80763, M80833), 2) small cell carcinoma (M80403 - 80453), 3) adenocarcinoma (M82303 -823133, M82503 - 82603, M84803 - 84903, M85503 - 85603, M85703 - 85723, M81403, M82113, M83233) 4) large cell carcinoma (M80123 -80313, M83103) and 5) other carcinomas (M85603, M82403, M82493, M84303, M82003, M85623, M80463) and unclassified malignant cells (M80013, M80103, M82463, M99903, M99993).

In the second part of the study outcome was time to death.

### Statistics

Differences in characteristics between groups were evaluated by the χ^2 ^test and Kruskal-Wallis Test when appropriate.

We estimated the probability of lung cancer and the relative risk of lung cancer in all study populations. Observation time was calculated from the respective index date to date of diagnosis of lung cancer or other cancers, death, 1 January 2009, emigration or lost to follow-up, whichever came first. We used cumulative incidence function to illustrate time to first lung cancer, recognizing death and diagnoses of other cancers as a competing risk [18]. Incidence rate ratios (IRR) and 95% confidence intervals for lung cancer as estimates of relative risks were calculated using Cox proportional-hazards regression. In the comparison of HIV patients and their matched population controls we used Cox regression analyses stratified according to the initial match criteria (age and gender).

In order to identify risk factors for lung cancer in the HIV population, we calculated incidence rate ratios (IRRs) stratified by gender, race (native vs. immigrant), age at index date (≤ 50 years vs. > 50 years), diagnosis of HIV before 1 January 1995, smoking (smoker or former smoker in any quantity vs. never smoked), route of infection (men who have sex with men (MSM) vs. heterosexually infected men vs. heterosexually infected women vs. injection drug user (IDU)), immunodeficiency at index date (AIDS defining event or CD4 cell count < 350 cells/μL vs. no AIDS defining event and CD4 cell count ≥ 350 cells/μL). All results were adjusted for age (continuous variable) and gender. Baseline CD4 cell count was defined as the measurement closest to index date +/- one year. Also, we included first date with a CD4 count below 350/μL or date of first AIDS defining event as a time dependent variable.

IRRs for parent populations were adjusted for age at parent index date (continuous variable) as well as year of birth of the parent divided into the following decades: - 1920, 1920-1930, 1930-1940, 1940-1950, 1950 - later. A robustness analysis was performed where we calculated IRRs for lung cancer including only parents of HIV patients and population controls born in Denmark.

In a sub analysis, we calculated IRR for the following four types of lung cancers: Squamous cell carcinoma, small cell carcinoma, adenocarcinoma and large cell carcinoma.

We computed person-years at risk of dying from lung cancer from date of the diagnosis to date of death, 1 January 2009, emigration or lost to follow up whichever came first. We used Kaplan-Meier analysis to construct survival curves and Cox regression analyses were used to estimate mortality rate ratios (MRR). In the population consisting of HIV patients and population controls all results were adjusted for age at lung cancer diagnosis (continuous variable) and gender. In the parent population all results were adjusted for age at lung cancer diagnosis (continuous variable) as well as year of birth of the parent divided into the following decades: - 1920, 1920-1930, 1930-1940, 1940-1950, 1950 - later. The study was approved by the Danish Data Protection Agency. SPSS statistical software, Version 15.0 (Norusis; SPSS Inc., Chicago, Illinois, USA) and R software, version 2.8.1, was used for data analysis.

## Results

In the first part of the study, we identified a total of 5,168 Danish HIV patients of which 115 were excluded due to cancer diagnosed prior to index date, leaving 5,053 HIV patients and 50,530 age and gender matched population controls in the study with a total of 37,622 and 470,322 person-years of follow-up, respectively (figure 1). 1,165 (23.1%) died during follow-up, 17 (0.3%) were lost to follow-up and 173 (3.4%) emigrated. 85.8% of the HIV patients were male, 71.6% were native Danes and 45.3% were MSMs. More patient characteristics are described in table 1. 71.1% of the HIV study population were smokers or former smokers in any quantity and females and immigrant were less likely to smoke whereas almost all IDUs smoked or used to smoke (Additional file 2).

**Table 1:** Characteristics of HIV patients and population controls with lung cancer

	HIV patients N = 5053,	HIV patients with lung cancer N = 29,	Population controls with lung cancer N = 183,	P value*
	N (%)	N (%)	N (%)	
Male gender	3827 (75.7)	27 (93.1)	156 (85.2)	0.253
Age at time of index date, median (IQR), years	36.6 (30.4 - 44.3)	49.9 (42.7 - 59.7)	50.7 (45.1 - 59.5)	0.554
Age at time of cancer diagnosis, median (IQR), years	-	57.0 (48.8 - 63.2)	58.9 (52.2 - 67.5)	0.192
Native Danes	3619 (71.6)	24 (82.8)	179 (97.8)	> 0.001
Older than 50 years at index date	695 (13.8)	14 (58.3)	98 (53.6)	0.129
Diagnosed with HIV before 1 January 1995	1968 (38.9)	18 (62.1)		
Smoker or former smoker in any quantity	2600 (71.1)	29 (100)		
Route of HIV infection				
Men who have sex with men	2287 (45.3)	12 (41.4)		
Heterosexually infected men	934 (18.5)	12 (41.4)		
Heterosexually infected women	930 (18.4)	2 (6.9)		
Injection drug user	553 (10.9)	3 (10.3)		
Other	349 (6.9)	0 (0.0)		
Baseline CD4 < 350 cells/μL or AIDS defining event at index date	2655 (52.5)	20 (69.0)		
Baseline CD4 cell count, cells/μL, median (IQR)	279 (104 - 480)	230 (86 - 403)		

**Histological classification of lung cancer**		**HIV patients with lung cancer** **N = 29**	**Population controls with lung cancer** **N = 183**	**P value**

Squamous cell carcinoma		8 (27.6)	36 (19.7)	
Small cell carcinoma		4 (13.8)	31 (16.9)	
Adenocarcinoma		7 (24.1)	55 (30.1)	
Large cell carcinoma		0 (0)	12 (6.5)	
Other and unclassified		10 (34.5)**	49 (26.8)***	
**P value for difference in distribution of HPV site, potential HPV site and potentially unrelated HPV site**				0.457

*P value for differences in characteristics between HIV patients and population controls with lung cancer

**3 carcinoma without other specifications (M80103), 3 Non-Large cell carcinomas (M80463), 2 adenosquamos carcinomas (M85603), 1 no microscopically confirmed lung cancer (M99903) and 1 malignant histology unknown (M99993).

***2 neoplasm malignant (M80003), 3 malignant tumor cells (M80013), 3 carcinoma without other specifications (M80103), 22 Non-Large cell carcinomas (M80463), 3 carcinoid tumor, malignant (M82403), 1 neuroendocrine carcinoma (M82463), 1 adenosquamous carcinomas (M85603), 12 not microscopically confirmed lung cancer (M99903) and 2 malignant histology unknown (M99993).

A total of 333 (6.6%) HIV patients and 1328 (2.6%) population controls were diagnosed with a cancer in the observation period of whom 29 (0.6%) and 183 (0.4%), respectively, were diagnosed with lung cancer. Median age at time of lung cancer was not significantly different in the two cohorts. Their characteristics are listed in table 1. All HIV patients with lung cancer were smokers. 20 (69.0%) of the HIV patients were treated with HAART before diagnosed with lung cancer. Nadir CD4 cell count in the HIV patients diagnosed with lung cancer was 110 cells/μL (IQR; 45 cells/μL - 230 cells/μL) and median CD4 cell count at time of cancer diagnosis was 299 cells/μL (IQR; 130 cells/μL - 521 cells/μL). 17 (85.0%) of those in HAART were virally suppressed at time of lung cancer diagnose.

**Figure 2:**
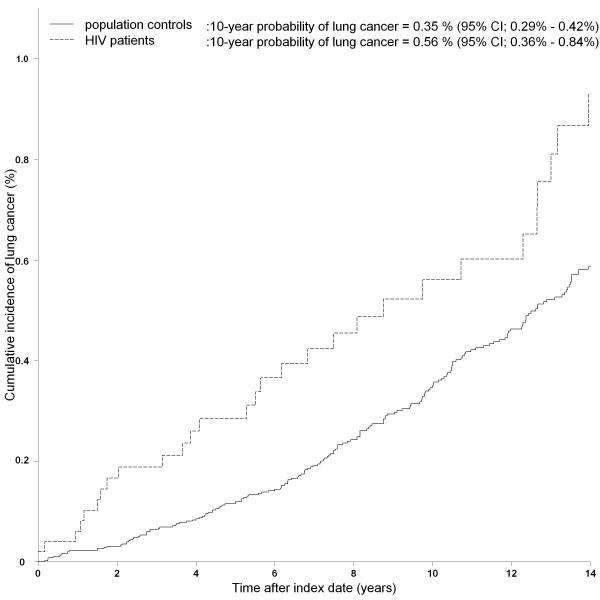
**Cumulative incidence function for lung cancer among HIV patients and population controls**.

As seen in figure 2, the HIV infected population had a higher 10-year probability for lung cancer compared to the control population. The incidence of lung cancer was higher in HIV patients than in population controls (adjusted IRR 2.38 (95% CI; 1.61 - 3.53)). The IRRs for lung cancer remained high in most stratified analyses except for female HIV patients. No events of lung cancer were diagnosed among HIV patients who never smoked (table 2). The highest risk of lung cancer were seen in HIV patients who were smokers or former smokers (adjusted IRR; 4.06 (95% CI; 2.66 - 6.21), male patients with heterosexual route of HIV infection (adjusted IRR; 4.19 (95% CI; 2.20 - 7.96)) and HIV patients with prior AIDS defining events or low baseline CD4 cell count (adjusted IRR; 3.25 (95% CI; 2.01 - 5.24)).

**Table 2:** Risk factors for lung cancer for HIV patients and population controls

	Lung cancer (HIV/population controls), N	Unadjusted IRR (95% CI)	Adjusted IRR* (95% CI)
All	29/183	2.08 (1.41 - 3.08)	2.38 (1.61 - 3.53)

**Stratification**			

Male gender*	27/156	2.33 (1.55 - 3.50)	2.66 (1.77 - 4.01)
Female gender	2/27	0.91 (0.22 - 3.82)	**
Native	24/179	2.13 (2.39 - 3.27)	2.56 (1.47 - 3.46)
Immigrant	5/4	6.38 (1.71 - 23.86)	**
Younger than or 50 years at index date	18/104	2.33 (1.35 - 4.04)	2.34 (1.35 - 4.06)
Older than 50 years at index date *	14/98	2.07 (1.18 - 3.63)	2.08 (1.19 - 3.65)
HIV diagnosed before 1995	18/112	2.34 (1.42 - 3.85)	2.60 (1.58 - 4.28)
HIV diagnosed after 1995 at index date	11/71	1.75 (0.93 - 3.31)	2.13 (1.12 - 4.03)
Smoker or former smoker in any quantity	29/81	3.79 (2.48 - 5.97)	4.06 (2.66 - 6.21)
Never smoked	0/35	**	**
Route of HIV infection			
Men who have sex with men (MSM)	12/99	1.60 (0.88 - 2.91)	1.82 (0.99 - 3.12)
Heterosexually infected men	12/44	3.40 (1.80 - 6.45)	4.19 (2.20 - 7.96)
Heterosexually infected women	2/18	1.26 (0.30 - 5.44)	**
Injection drug user (IDU)	3/13	1.95 (1.28 - 2.94)	**
Baseline CD4 < 350 cells/μL or AIDS defining event at index date	20/111	2.70 (1.68 - 4.35)	3.25 (2.01 - 5.24)
Baseline CD4 *≥ *350 cells/μL and no AIDS defining event at index date	6/40	1.68 (0.71 - 3.96)	1.71 (0.72 - 4.03)

* All adjusted for gender and age, except the stratification on male which is only adjusted for age and age > 50 which is only adjusted for gender.

** Too few events for analysis.

In the time period after first date with a CD4 cell count below 350 cells/μL or an AIDS defining event the adjusted RR for lung cancer was 1.48 (95% CI; 0.63 - 3.47)) compared to HIV patients with intact or restituted immune system.

**Figure 3:**
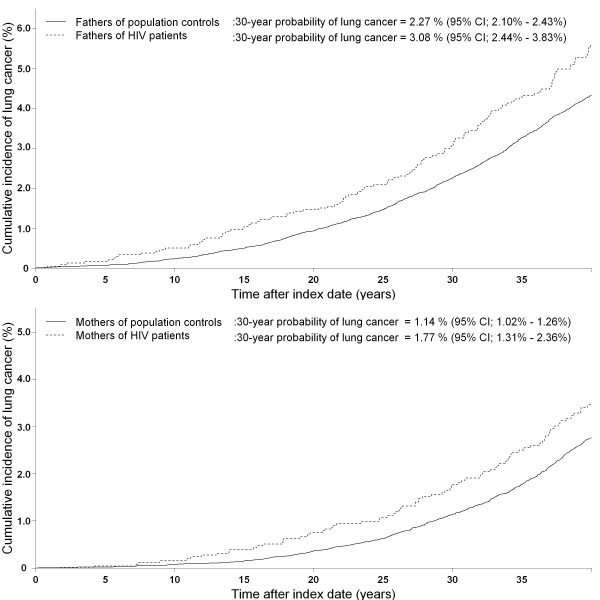
**Cumulative incidence function for lung cancer among parents of HIV patients and parents of population controls**.

We identified a total of 2,428 fathers of HIV infected individuals vs. 31,929 fathers of population controls and 2,586 mothers of HIV patients and 32,705 mothers of population controls (figure 1). Age at paternal index date and median follow-up time did not differ between the parent populations (table 3). The 30-year probability of lung cancer was higher for both fathers and mothers of HIV patients (figure 3). The adjusted relative risk for lung cancer for fathers and mothers of HIV patients was 1.31 (95% CI: 1.09 - 1.58) and 1.35 (95% CI: 1.07 - 1.70), respectively. After stratification of the children's route of HIV infection, the relative risk remained high in all groups particularly for parents of IDUs (adjusted IRR for HIV fathers; 1.63 (95% CI: 1.02 - 2.58), adjusted IRR for HIV mothers; 2.01 (95% CI: 1.21 - 3.36)) (table 4).

**Table 3:** Characteristics of parents of HIV patients and population controls

	Fathers of HIV patients	Fathers of population controls	Mothers of HIV patients	Mothers of population controls
	N, (%)	N, (%)	N, (%)	N, (%)
All	2,428	31,929	2,586	32,705
Median age at index date, years (IQR).	34.3 (28.6 - 41.9)	33.4 (28.3 - 40.6)	30.9 (25.8 - 37.5)	30.5 (25.7 - 36.7)
Follow-up time, median, years (IQR)	34.5 (25.2 - 40.0)	35.5 (27.6 - 40.0)	37.5 (29.0- 40.0)	38.4 (31.3 - 40.0)
Duration of follow-up, years	75,125	1,026,778	86,024	1,124,126
Emigration during follow-up, N (%)	41 (1.7)	327 (1.0)	31 (1.2)	219 (0.7)
Lost to follow-up, N (%)	4 (0.1)	13 (<0.1)	2 (0.1)	2 (<0.1)
Diagnosed with all lung cancers, (N (%))	122 (5.0)	1233 (3.9)	78 (3.0)	763 (2.3)
Median age at diagnose of all lung cancers, years (IQR)	65.7 (60.1 - 72.8)	65.6 (59.3 - 71.5)	60.4 (55.2 - 68.9)	64.1 (58.2 - 69.9)

**Table 4:** Incidence rate ratios for lung cancer for the parent population stratified for the offspring's route of HIV infection

	Cancer, N	Unadjusted IRR (95% CI)	Adjusted IRR* (95% CI)
All fathers of HIV patients**	122/1233	1.38 (1.14 - 1.66)	1.31 (1.09 - 1.58)

**Stratification**			

**Route of HIV infection for offspring**			
Men who have sex with men (MSM)	65/535	1.34 (1.04 - 1.73)	1.24 (0.96 - 1.61)
Heterosexually infected	31/440	1.39 (0.97 - 2.00)	1.31 (0.91 - 1.89)
Injection drug user (IDU)	20/183	1.40 (0.89 - 2.23)	1.63 (1.02 - 2.58)

All mothers of HIV infected***	78/763	1.37 (1.08 - 1.72)	1.35 (1.07 - 1.70)

**Stratification**			

**Route of HIV infection for offspring**			
Men who have sex with men (MSM)	41/342	1.29 (0.93 - 1.78)	1.26 (0.90 - 1.73)
Heterosexually infected	17/262	1.20 (0.73 - 1.96)	1.20 (0.73 - 1.96)
Injection drug user (IDU)	17/112	1.84 (1.10 - 3.06)	2.01 (1.21 - 3.36)

* Adjusted for age at parent index date (continuous variable) as well as year of birth of the parent divided into the following decades: - 1920, 1920-1930, 1930-1940, 1940-1950, 1950 - later.

** There were five cases of lung cancer among fathers of children who had other route of HIV infection and 85 among fathers of population controls.

*** There were one case of lung cancer among mothers of children who had other route of HIV infection

### Sub-analyses

In a sub analysis including only squamous cell carcinoma, small cell carcinoma, adenocarcinoma and large cell carcinoma, 19 (0.4%) and 134 (0.3%) lung cancers where diagnosed in the HIV patients and population controls, respectively. The adjusted IRR was 2.11 (95% CI: 1.30 - 3.42). In the parent population, 85 (3.4%) vs. 965 (2.9%) lung cancers were diagnosed in the fathers and 60 (2.2%) vs. 597 (1.8%) in the mothers. The IRRs were increased in both parents of HIV patients (HIV fathers adjusted; 1.17 (95% CI: 0.93 -1.76), HIV mothers adjusted; 1.32 (95% CI: 1.01 -1.72)). Also in a robustness analysis including only parents of HIV patients and population controls born in Denmark, the relative risks of lung cancer were increased (HIV fathers adjusted; 1.39 (95% CI: 1.15 -1.68), HIV mothers adjusted; 1.35 (95% CI: 1.06 -1.72)).

### Mortality

**Figure 4:**
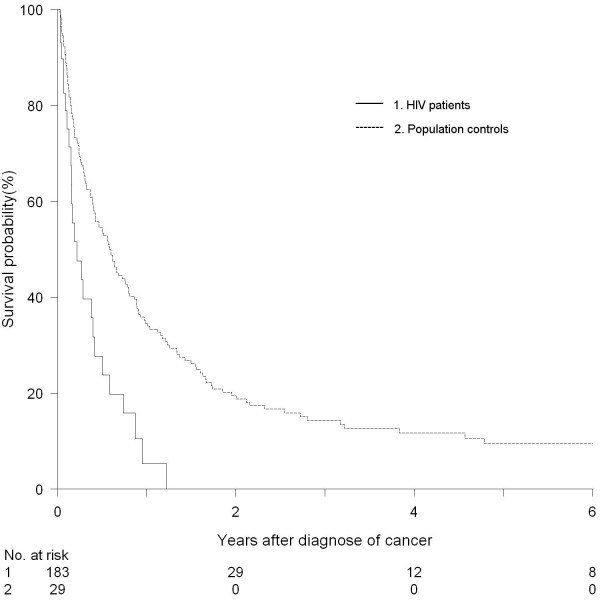
**Probability of survival after diagnose of lung cancer among HIV patients and population controls**.

25 HIV patients and 149 population controls with lung cancer died during follow-up. Median survival time for the HIV infected population was approximately two months and seven months for the population controls (figure 4). Adjusted MRR for the HIV patients with lung cancer was 2.33 (95%CI; 1.51 - 3.51).

Survival in the parent populations did not differ between parents of HIV patients and population controls (HIV fathers adjusted MRR; 0.97 (95%CI; 0.80 - 1.18), HIV mothers adjusted; 0.96 (95%CI; 0.75 - 1.23).

## Discussion and Conclusion

We found a more than twofold increased risk of lung cancer in HIV patients compared with the background population. The risk was especially increased in HIV patients who were smokers or former smokers, heterosexually infected men or immunosuppressed. Both fathers and mothers of HIV patients had an increased risk of lung cancer compared to the parents of the non-HIV infected population controls. All HIV patients diagnosed with lung cancer were smokers or former smokers and HIV appeared to be a marker of behavioural or family related risk factors that affect the incidence of lung cancer independently of immunosuppression. Also the risk of death was more than twofold increased in the HIV infected population with lung cancer compared to the background population. The increased risk of death in patients with lung cancer appears to be related to the HIV infection since parents of HIV infected with lung cancer did not have an increased mortality.

To our knowledge this is the first nationwide study comparing the incidence of lung cancer in HIV patients with that of a matched control population. A major strength of the study is the quality and coverage of the Danish registries with access to valid data on family members and the population-based design with long and nearly complete follow-up.

We did not estimate the risk of lung cancer and death according to the implementation of HAART. However, the development of lung cancer presumably takes several years and it is therefore difficult to estimate the impact of risk factors that vary over time. Our registry data did not allow direct adjustment for potential effects of confounders like smoking. Therefore we chose to include the parent population in order to demonstrate a family related risk associated with lung cancer as an indirect measure of risk behaviour. Our cohort had a size that allowed us to detect the risk of rare outcomes such as lung cancer. The small number of cases caused some imprecision of our estimates and did not allow us to perform complex multivariate analyses. Our study is based on data extracted from registries and misclassification cannot be ruled out. Still, calculations performed on specific histologically confirmed lung cancer diagnoses confirmed the increased risk in HIV patients. We were not able to include data on lung cancer staging in the mortality analysis which could confound our results. Finally, a limitation of the study is the difference in the proportion of traceable parents among HIV patients and population controls due to a larger proportion of immigrants among the HIV patients. However, a robustness analysis including only parents of HIV patients and population controls born in Denmark confirmed the increased risk of lung cancer among parents of HIV patients.

We observed a twofold increased risk of lung cancer in the HIV infected population which corresponds to the findings of other studies that estimated standardized incidence ratios on population based material [5,19,20]. All lung cancers are related to smoking, especially squamous cell carcinoma and small cell carcinoma [21]. We found a high prevalence of smoking among the Danish HIV patients, where little less than 30% never smoked compared to 40% among adults in the general background population in Denmark in the period 1987 - 2005 [22]. Accordingly, HIV patients who were smokers or former smokers had a considerably increased risk of lung cancer whereas no cases of lung cancer occurred among non-smoking HIV patients. The risk of lung cancer was low for women with HIV but considerably increased for HIV infected males with heterosexual route of infection, HIV patients with previous AIDS defining events or low CD4 cell count in both the stratified and time-update analysis. In the general population the risk of lung cancer is lower in females corresponding to our finding but the estimate was based on few events [23]. The relative risk was considerably increased in male patients with heterosexual route of infection although the prevalence of smoking did not differ compared to homosexually infected individuals. Guiguet et al found an inverse correlation between risk of lung cancer and decreasing CD4 cell count [24]. In accordance with this and other studies we found that immunosuppression predicts risk of lung cancer [8]. The mechanism behind this is unknown but although patients with immunodeficiency at time of inclusion did not smoke more than the rest of the HIV infected population, immunodeficiency may accentuate the oncogenic effects of smoking. Corresponding to the findings of other studies we found no difference in the distribution of histopathological sub-types of lung cancer in the two populations pointing towards a common set of risk factors (probably smoking) [10,11].

Also parents of HIV patients in general and in particular parents of IDUs have an excess risk of lung cancer implying that family associated or behavioral risk factors are involved. Smoking is related to risk-taking behaviour which is moderately to strongly related to heritability and offspring of smokers have a four times higher risk of initiating smoking [25,26]. Several studies has been conducted on this subject and concerning smoking a social learning model of smoking initiation has been demonstrated where adolescents imitate the behaviour of adults over time with repeated exposure [27]. Knowing that the prevalence of smoking is high in the Danish HIV patients it seems likely that smoking explains a substantial part of the increased risk of lung cancer in both the HIV patients and parents. HIV therefore appears to be a marker of family related risk factors that affect the incidence of lung cancer.

In concordance with other studies HIV patients diagnosed with lung cancer had an increased mortality compared to the population controls [10-12]. This result may be confounded by an increased competing risk of death in the HIV infected population or differences in staging of lung cancer, which we were not able to adjust for. No difference in mortality was seen in the parent population suggesting that the decreased survival in HIV patients is related to HIV and not family related risk factors.

In conclusion, the risk was especially increased in HIV patients who were smokers or former smokers, heterosexually infected men or immunosuppressed. The higher risk of lung cancer in both HIV patients and their parents suggest that family associated risk factors, like tobacco use, explain part of the increased risk in HIV patients. HIV appears to be a marker of behavioural or family related risk factors that affect the risk of lung cancer. The use of tobacco is the major risk factor for lung cancer and future interventions should focus on cessation and preventing initiation of smoking.

## Competing interests

NO has received research funding from Roche, Bristol-Myers Squibb, Merck Sharp & Dohme, GlaxoSmithKline, Abbott, Boehringer Ingelheim, Janssen-Cilag, Swedish Orphan and NOVO Nordisk Foundation. FE has received research funding from Merck Sharp & Dohme. JG has received research funding from Abbott, Roche, Bristol-Myers Squibb, Merck Sharp & Dohme, Pharmasia, GlaxoSmithKline, Swedish Orphan and Boehringer Ingelheim. CP has received funding from Abbott, Roche, Bristol-Myers Squibb, Merck Sharp & Dohme, GlaxoSmithKline, Swedish Orphan and Boehringer Ingelheim.

GK, CSL and GP have no conflicts of interest.

## Authors' contributions

The authors contributions are the following: FNE (MD) contributed with study design, data collection, data analysis, interpretation of findings and writing of the manuscript. GK (MD, associate professor, DrMedSc), CSL (MD, DrMedSc), GP (MD) and CP (MD, professor, DrMedSc) contributed with data collection, study design, interpretation of findings and critical edit of the manuscript. JG (MD, Professor, DrMedSc) contributed with study design, data collection, interpretation of findings and critical edit of the manuscript. NO (MD, DrMedSc) contributed with data collection, study design, critical review of data analyses, interpretation of findings and critical edit of the manuscript. All authors read and approved the final manuscript.

## Pre-publication history

The pre-publication history for this paper can be accessed here:

http://www.biomedcentral.com/1471-2407/11/272/prepub

## Supplementary Material

Additional file 1 **Cancer definitions**. ICD-7 and ICD-10 cancer definitions used in the study. Click here for file

Additional file 2 **Smokers or former smokers in Danish HIV infected**. Distribution of smokers or former smokers in Danish HIV infected. Click here for file

## References

[B1] Lohse N Hansen AB Pedersen G Kronborg G Gerstoft J Sorensen HT Vaeth M Obel N Survival of persons with and without HIV infection in Denmark, 1995-2005 Ann Intern Med 2007 146 2 87 95 17227932 10.7326/0003-4819-146-2-200701160-00003

[B2] Engels EA Biggar RJ Hall HI Cross H Crutchfield A Finch JL Grigg R Hylton T Pawlish KS McNeel TS Goedert JJ Cancer risk in people infected with human immunodeficiency virus in the United States Int J Cancer 2008 123 1 187 94 10.1002/ijc.23487 18435450

[B3] Patel P Hanson DL Sullivan PS Novak RM Moorman AC Tong TC Holmberg SD Brooks JT Incidence of types of cancer among HIV-infected persons compared with the general population in the United States, 1992-2003 Ann Intern Med 2008 148 10 728 36 18490686 10.7326/0003-4819-148-10-200805200-00005

[B4] Silverberg MJ Chao C Leyden WA Xu L Tang B Horberg MA Klein D Quesenberry CP Jr Towner WJ Abrams DI HIV infection and the risk of cancers with and without a known infectious cause AIDS 2009 23 17 2337 45 10.1097/QAD.0b013e3283319184 19741479 PMC2863991

[B5] Chaturvedi AK Pfeiffer RM Chang L Goedert JJ Biggar RJ Engels EA Elevated risk of lung cancer among people with AIDS AIDS 2007 21 2 207 13 10.1097/QAD.0b013e3280118fca 17197812

[B6] Engels EA Brock MV Chen J Hooker CM Gillison M Moore RD Elevated risk of lung cancer among HIV-infected individuals J Clin Oncol 2006 24 9 1383 8 10.1200/JCO.2005.03.4413 16549832

[B7] Giordano TP Kramer JR Does HIV infection independently increase the incidence of lung cancer? Clin Infect Dis 2005 40 3 490 1 10.1086/427028 15668878

[B8] Grulich AE van Leeuwen MT Falster MO Vajdic CM Incidence of cancers in people with HIV/AIDS compared with immunosuppressed transplant recipients: a meta-analysis Lancet 2007 370 9581 59 67 10.1016/S0140-6736(07)61050-2 17617273

[B9] Lavole A Chouaid C Baudrin L Wislez M Raguin G Pialoux G Girard PM Milleron B Cadranel J Effect of highly active antiretroviral therapy on survival of HIV infected patients with non-small-cell lung cancer Lung Cancer 2009 65 3 345 50 10.1016/j.lungcan.2008.11.018 19135758

[B10] Tirelli U Spina M Sandri S Serraino D Gobitti C Fasan M Sinicco A Garavelli P Ridolfo AL Vaccher E Lung carcinoma in 36 patients with human immunodeficiency virus infection. The Italian Cooperative Group on AIDS and Tumors Cancer 2000 88 3 563 9 10.1002/(SICI)1097-0142(20000201)88:3<563::AID-CNCR11>3.0.CO;2-D 10649248

[B11] Sridhar KS Flores MR Raub WA Jr Saldana M Lung cancer in patients with human immunodeficiency virus infection compared with historic control subjects Chest 1992 102 6 1704 8 10.1378/chest.102.6.1704 1446476

[B12] Brock MV Hooker CM Engels EA Moore RD Gillison ML Alberg AJ Keruly JC Yang SC Heitmiller RF Baylin SB Herman JG Brahmer JR Delayed diagnosis and elevated mortality in an urban population with HIV and lung cancer: implications for patient care J Acquir Immune Defic Syndr 2006 43 1 47 55 10.1097/01.qai.0000232260.95288.93 16936558

[B13] The Central Office of Civil Registration The civil Registration System in Denmark 2011 http://www.cpr.dk/cpr/site.aspx?p=198&areaid=27&ArticleTypeID=76&t=ForsideVisartikel&Articleid=

[B14] Obel N Engsig FN Rasmussen LD Larsen MV Omland LH Sorensen HT Cohort profile: the Danish HIV cohort study Int J Epidemiol 2009 38 5 1202 6 10.1093/ije/dyn192 18799495

[B15] Storm HH Michelsen EV Clemmensen IH Pihl J The Danish Cancer Registry--history, content, quality and use Dan Med Bull 1997 44 5 535 9 9408738

[B16] NORDCAN 2010 http://www-dep.iarc.fr

[B17] Beasley MB Brambilla E Travis WD The 2004 World Health Organization classification of lung tumors Semin Roentgenol 2005 40 2 90 7 10.1053/j.ro.2005.01.001 15898407

[B18] Marubini E Valsecci MG Analysing Survival Data from Clinical trials and Observational Studies, 1 st ed Chichester, England: John Wiley & Sons 1995 331 63

[B19] Powles T Robinson D Stebbing J Shamash J Nelson M Gazzard B Mandelia S Moller H Bower M Highly active antiretroviral therapy and the incidence of non-AIDS-defining cancers in people with HIV infection J Clin Oncol 2009 27 6 884 90 10.1200/JCO.2008.19.6626 19114688

[B20] Herida M Mary-Krause M Kaphan R Cadranel J Poizot-Martin I Rabaud C Plaisance N Tissot-Dupont H Boue F Lang JM Costagliola D Incidence of non-AIDS-defining cancers before and during the highly active antiretroviral therapy era in a cohort of human immunodeficiency virus-infected patients J Clin Oncol 2003 21 18 3447 53 10.1200/JCO.2003.01.096 12972519

[B21] Khuder SA Mutgi AB Effect of smoking cessation on major histologic types of lung cancer Chest 2001 120 5 1577 83 10.1378/chest.120.5.1577 11713137

[B22] Ekholm O Kjøller M Davidsen M Hesse U Eriksen L Christensen AI Grønbæk M Sundhed og sygelighed i Danmark & udviklingen siden 1987 2006 Statens Institut for Folkesundhed. København http://www.si-folkesundhed.dk [In Danish]

[B23] Skuladottir H Olsen JH Hirsch FR Incidence of lung cancer in Denmark: historical and actual status Lung Cancer 2000 27 2 107 118 10.1016/S0169-5002(99)00104-X 10688493

[B24] Guiguet M Boue F Cadranel J Lang JM Rosenthal E Costagliola D Effect of immunodeficiency, HIV viral load, and antiretroviral therapy on the risk of individual malignancies (FHDH-ANRS CO4): a prospective cohort study Lancet Oncol 2009 10 12 1152 9 10.1016/S1470-2045(09)70282-7 19818686

[B25] Marvin Zuckerman Michael Kuhlman D Personality and Risk-Taking: Common Bisocial Factors Journal of Personality 2010 68 6 999 1029 10.1111/1467-6494.00124 11130742

[B26] den Exter Blokland EA Engels RC Hale WW III Meeus W Willemsen MC Lifetime parental smoking history and cessation and early adolescent smoking behavior Prev Med 2004 38 3 359 68 10.1016/j.ypmed.2003.11.008 14766120

[B27] Gilman SE Rende R Boergers J Abrams DB Buka SL Clark MA Parental smoking and adolescent smoking initiation: an intergenerational perspective on tobacco control Pediatrics 2009 123 2 e274 e281 10.1542/peds.2008-2251 19171580 PMC2632764

