# Supplementary material for: Lung cancer in HIV patients and their parents: A Danish cohort study
Source: BMC Cancer. 2011 Jun 25;11:272. doi: 10.1186/1471-2407-11-272 (PMC3135571; doi:10.1186/1471-2407-11-272)
Supplement: Additional file 1 — Cancer definitions. ICD-7 and ICD-10 cancer definitions used in the study. [file 1471-2407-11-272-S1.DOC]

Cancers included as competing risk in the study were coded according to The International Classification of Diseases (ICD) version 7 in the period 1969 – 1977 and ICD-10 in the period 1978 – 2009: Lib cancer (ICD-7; 1400 – 1402, ICD-10; C00), Tongue cancer (ICD-7; 1410 – 1418, ICD-10; C01 - C02), Oral cavity cancer (ICD-7; 1430 – 1441, ICD-10 ; C03- C06), Salivary glands cancer (ICD-7; 1420 – 1423, ICD-10; C07 - C08), Tonsil cancer (ICD-7; 1450, ICD-10; C09), Pharynx cancer (ICD-7; 1451 – 1458, 1470 - 1480, ICD-10; C10), Sinuses, nasal cavity and middle ear (ICD-7; 1600 – 1602, ICD-10; C30), Larynx cancer (ICD-7; 1610– 1611, ICD-10; C32), Trachea cancer (ICD-7; 2620, ICD-10;C33), Pleura cancer (ICD-7; 1622, ICD-10; C384, C45), Oesophagus, stomach and small intestine cancer (ICD-7; 1500 – 1522, ICD-10; C15 - C17), Colon cancer (ICD-7; 1530 – 1535, ICD-10; C18 - C20), Anal cancer (ICD-10; C21), Pancreas cancer (ICD-7; 1570, ICD-10; C25), Liver cancer (ICD-7; 1550, ICD-10; C22), Biliary tract cancer (ICD-7; 1551 – 1553, ICD-10;C23, C24), Lymphoid cancer (ICD-7; 2000 - 2044, ICD-10; C81 - C85, C90 - C96), Urinary tract cancers (ICD-7; 1800 – 1818, ICD-10; C64 - C68, D90, D414), Skin cancer (ICD-7; 1900, ICD-10; C43, C46), Bone, articular cartilage, mesothelial and soft tissue cancer (ICD-7; 1960 – 1979, ICD-10; C40, C41, C46.1 C49), Breast cancer (ICD-7; 1700 – 1705, ICD-10;C50), Female genital cancer (ICD-7; 1710 – 1769, ICD-10; C51 -C58), Eye, brain and other parts of the central nerve system (ICD-7; 1920 – 1933, ICD-10; C69 - C72, D32, D34, D42, D43), Male genital organs (ICD-7; 1770 – 1793, ICD-10; C60 - C63), Thyroid and other endocrine organs (ICD-7; 1940 – 1954, ICD-10; C73 - C75), Kaposi sarcoma (ICD-10; C46).
