# Supplementary material for: Lung cancer in HIV patients and their parents: A Danish cohort study
Source: BMC Cancer. 2011 Jun 25;11:272. doi: 10.1186/1471-2407-11-272 (PMC3135571; doi:10.1186/1471-2407-11-272)
Supplement: Additional file 2 — Smokers or former smokers in Danish HIV infected. Distribution of smokers or former smokers in Danish HIV infected. [file 1471-2407-11-272-S2.DOC]

| **Characteristics** | **N** | **Smoker or former smoker, N (%)** | **Patients asked about smoking, N (%)** |
| --- | --- | --- | --- |
| All | 5053 | 2600 (71.1) | 3658 (72.4) |
| Male gender | 3827 | 2069 (75.6) | 2736 (71.5) |
| Female gender | 1226 | 531 (57.6) | 922 (75.2) |
| Native | 3619 | 2008 (76.4) | 2628 (72.6) |
| Immigrant | 1434 | 592 (57.5) | 1030 (71.8) |
| Younger than 50 years | 4358 | 2247 (71.0) | 3167 (72.7) |
| Older than 50 years at index date | 695 | 353 (71.9) | 491 (70.6) |
| Route of HIV infection |  |  |  |
| Men who have sex with men | 2287 | 1280 (74.1) | 1727 (75.1) |
| Heterosexually infected men | 934 | 502 (74.0) | 678 (72.6) |
| Heterosexually infected women | 930 | 365 (50.4) | 724 (77.8) |
| Injection drug user | 553 | 325 (97.6) | 333(60.2) |
| Other or unknown | 349 | 128 (65.3) | 196 (56.2) |
| Diagnosed with HIV before 1 January 1995 | 1968 | 948 (77.3) | 1226 (62.3) |
| Diagnosed with HIV after 1 January 1995 | 3085 | 1652 (67.9) | 2432 (78.8) |
| Baseline CD4 >= 350 cells/µL and no AIDS defining event at index date | 1619 | 1216 (66.3) | 1308 (80.8) |
| Baseline CD4 < 350 cells/µL or AIDS defining event at index date | 2655 | 991 (75.8) | 1834 (69.1) |
|  |  |  |  |
|  |  |  |  |
